# Rapidly Progressive Glomerulonephritis Due to IgA Nephropathy in a Patient With a Large Renal Mass

**DOI:** 10.7759/cureus.76015

**Published:** 2024-12-19

**Authors:** David Shi, Mona Ghias, Kevin Bogdansky, Asif Haris

**Affiliations:** 1 Internal Medicine, West Virginia University, Morgantown, USA; 2 Nephrology, West Virginia University, Morgantown, USA

**Keywords:** glomerulonephritis, igan, iga nephropathy, renal cell carcinoma, renal mass

## Abstract

IgA nephropathy (IgAN) is a common primary glomerulonephritis characterized by the deposition of IgA immune complexes within the glomerular mesangium. IgAN can present with a wide range of clinical manifestations, ranging from asymptomatic hematuria to severe renal disease. This case describes a 67-year-old woman with a history of diabetes mellitus, hypertension, and obesity who presented with acute kidney injury and clinical manifestations of nephrotic syndrome. A renal biopsy confirmed the diagnosis of IgAN. Additionally, imaging studies revealed a large, complex renal mass, raising concerns for renal cell carcinoma. The IgAN was treated with high-dose corticosteroids; however, the patient opted for active surveillance of the renal mass rather than surgical intervention. This case highlights the complex clinical presentation of IgAN and the challenges associated with managing patients with both IgAN and renal mass.

## Introduction

IgA nephropathy (IgAN) is the most common primary glomerulonephritis worldwide [1]. It is characterized by the deposition of IgA immune complexes within the glomerular mesangium [2]. IgAN can present with a variety of clinical manifestations, including hematuria, proteinuria, and acute kidney injury (AKI), with variable severity. Mild presentations typically involve incidental findings of microscopic hematuria with or without proteinuria in asymptomatic patients [3]. Young patients often present with recurrent gross hematuria following an upper respiratory tract infection (synpharyngitic hematuria), which rarely progresses to chronic kidney disease [4]. In more severe cases, IgAN can manifest as acute glomerulonephritis or rapidly progressive glomerulonephritis, accompanied by AKI, anasarca, hypertension, proteinuria, and hematuria [5].

The pathogenesis of primary IgAN is not well-defined; however, current theories suggest that defective IgA1-producing cells lead to under-galactosylation of the hinge region of IgA1. Antibodies targeting galactose-deficient IgA1 form immune complexes that deposit in the glomerulus. Mesangial cells exposed to these immune complexes release factors that promote inflammation and sclerosis [6,7]. Diagnosis of IgAN is established through kidney biopsy, which typically reveals mesangial proliferation with IgA immune deposits on immunofluorescence microscopy [8].

Here, we present the case of a 67-year-old woman with progressively worsening renal function and a large kidney mass, ultimately diagnosed with biopsy-confirmed IgAN. The suspicious imaging features of the renal mass raised concern for renal cell carcinoma (RCC), highlighting the importance of considering malignancy and its potential paraneoplastic effects in such presentations.

## Case presentation

A 67-year-old woman with a history of type 2 diabetes, hypertension, and obesity presented to the emergency department on referral from her primary care physician due to worsening renal function noted on outpatient labs. She reported a three-month history of fatigue, leg swelling, and intermittent episodes of dark brown urine. On admission, she was hypertensive with a blood pressure of 172/65 mmHg. Laboratory studies revealed elevated creatinine and blood urea nitrogen (BUN), reduced estimated glomerular filtration rate (eGFR), hypoalbuminemia, and nephrotic-range proteinuria (Table 1). Urinalysis showed significant proteinuria and hematuria.

**Table 1:** Labs during hospital stay BUN: blood urea nitrogen, eGFR: estimated glomerular filtration rate, Cr: creatinine, HIV: human immunodeficiency virus, ANCA: antineutrophil cytoplasmic antibodies, C3: component 3, C4: component 4, DNA: deoxyribonucleic acid, Ab: antibody, ANA: antinuclear antibody, FLC: free light chain, TP: treponema pallidum

Lab value	Normal range	Initial days of admission	Close to discharge
Hemoglobin	11.5-16.0 g/dl	8.9 g/dl	8.6 g/dl
BUN	8-25 mg/dl	28 mg/dl	72 mg/dl
Creatinine	0.49-1.10 mg/dl	4.40 mg/dl	2.83 mg/dl
eGFR	>60 mL/min/1.73mˆ2	10	20
Random urine protein/Cr ratio	10-105 mg/g	15,963	N/A
HIV	Negative	Negative	N/A
Hepatitis B core antibody	Negative	Negative	N/A
Hepatitis B surface antibody	Negative	Negative	N/A
ANCA screen	Negative	Negative	N/A
C3 complement	81-157 mg/dl	112 mg/dl	N/A
C4 complement	12-39 mg/dl	30 mg/dl	N/A
Cryoglobulin	Negative	Negative	N/A
Anti-DNA antibody	Negative	Negative	N/A
Hepatitis C antibody	Negative	Negative	N/A
Phospholipase A2 receptor Ab	<14 RU/ml	<4 RU/ml	N/A
ANA	Negative	Positive	N/A
ANA titer	N/A	1:640	N/A
Glomerular basement membrane Ab	Negative	Negative	N/A
Kappa/Lambda FLC ratio	0.80-2.10	1.28	N/A
Syphilis TP Ab	Non-reactive	Non-reactive	N/A

**Figure 1:**
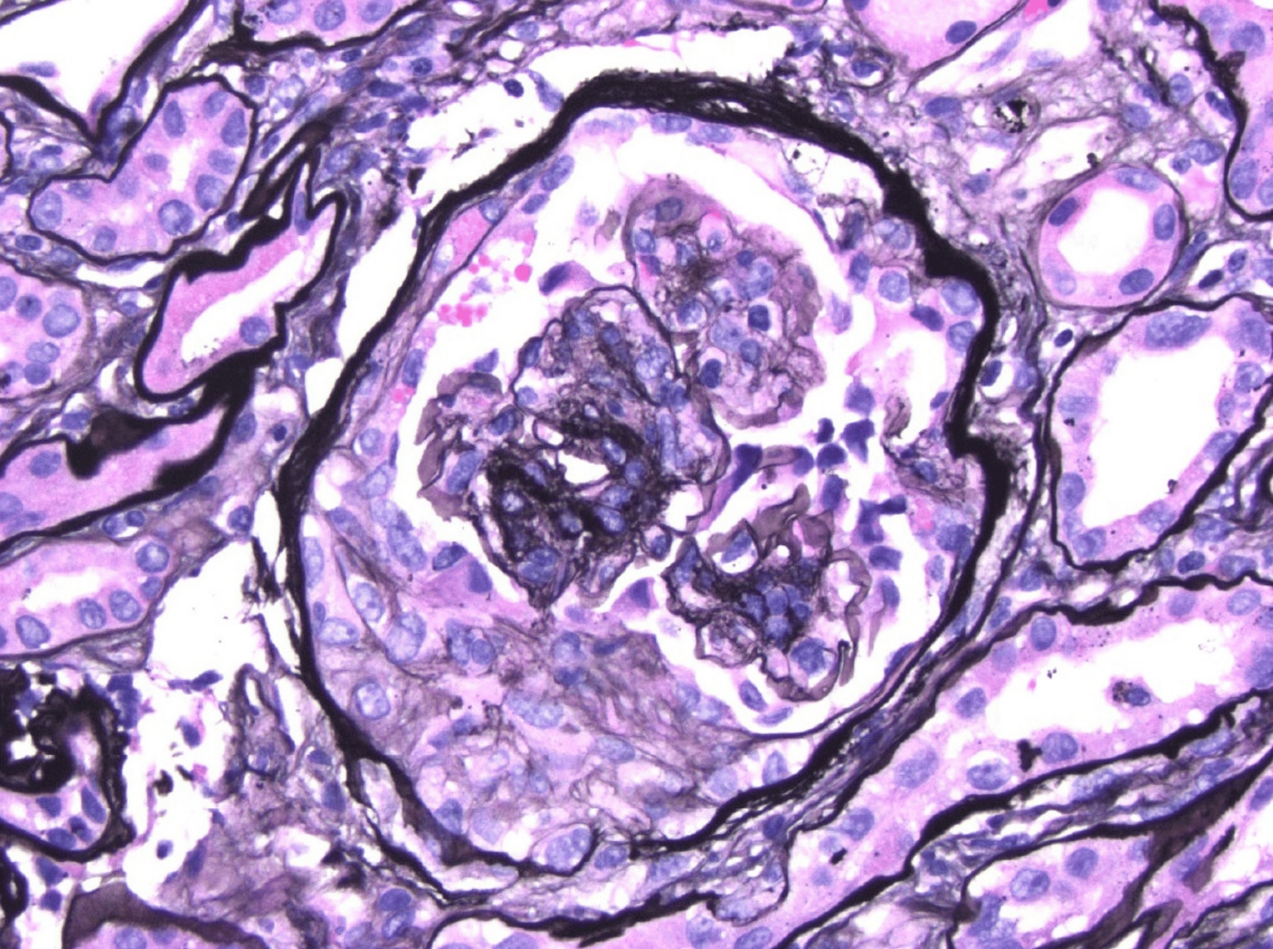
Light microscopy showing crescent formation

**Figure 2:**
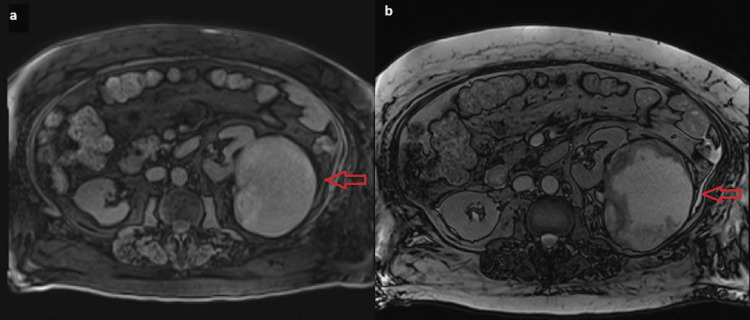
T1 (a) and T2 (b) MRI of the left kidney mass indicated by arrows MRI: magnetic resonance imaging

**Figure 3:**
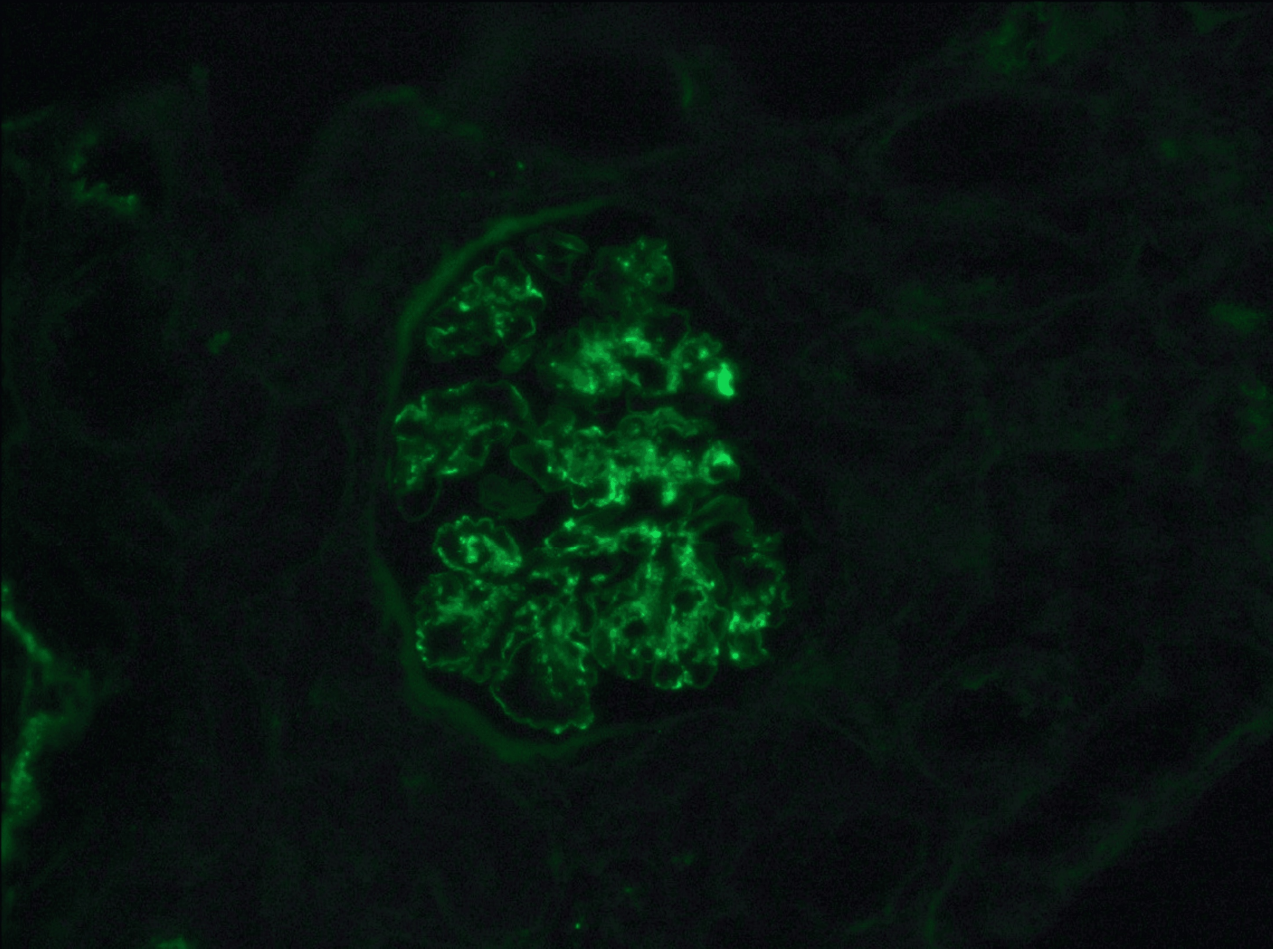
Immunofluorescence shows IgA deposition in the mesangium IgA: immunoglobulin A

Further workup for nephrotic-range proteinuria included negative anti-GBM antibodies, c-ANCA/p-ANCA antibodies, and anti-PLA2R antibodies. Complement levels C3 and C4 were within the range of normal, and infectious testing was unremarkable. Imaging with renal ultrasound revealed a large complex cystic mass arising from the mid-left kidney. A renal biopsy of the right kidney confirmed IgAN, showing mesangial IgA and C3 deposition, occasional crescents, and tubulointerstitial inflammation (Figures 1-3).

The patient was treated with intravenous methylprednisolone followed by oral prednisone with a gradual taper. Her renal function improved, with creatinine trending downward, and she was discharged in stable condition.

For the left renal mass, further evaluation with renal MRI demonstrated suspicious features consistent with cystic RCC (Figure 2). A biopsy of the mass revealed necrotic tissue with no definitive evidence of malignancy. Cytological analysis of the cyst fluid showed inflammatory cells and debris. Following a multidisciplinary discussion at the tumor board, it was decided to manage the cystic mass with serial outpatient imaging.

## Discussion

IgAN can be seen in association most commonly with liver disease, infections, autoimmune disorders, and malignancies [9]. The actual prevalence of secondary IgAN due to an underlying disorder, however, is difficult to discern. No distinct histological finding differentiates primary from secondary IgAN. In addition, the prevalence of mesangial IgA deposits can be 4-16% in some parts of the world, thus making it difficult to determine what is secondary IgAN and what is a coincidental finding of primary IgAN [10].

Liver disease is the most common cause of secondary IgAN. Serum IgA levels can be two to four times higher in cirrhotic patients and are abnormally glycosylated. The hypergammaglobulinemia in cirrhotics may be due to overproduction or decreased clearance [9]. In cirrhotics, small intestinal bacterial overgrowth and translocation of bacteria across the gut lead to immune system activation and overproduction of immunoglobulins [11,12]. Decreased clearance of immunoglobulins by hepatocytes in cirrhotics is felt to be multifactorial from a combination of abnormal receptor expression on the surface of hepatocytes, decreased size of endothelial fenestrae, and portal hypertension with portosystemic shunting [9].

Infection has long been implicated as a potential trigger for secondary IgAN. The viruses frequently associated with IgAN include hepatitis B, hepatitis C, and HIV. Bacterial infections commonly associated with IgAN include staphylococcus and streptococcus mucosal infections, *Chlamydia pneumonia*, and Lyme disease [9].

While the most common malignancies associated with IgAN are in the lung and GI tract, there are ample reports of IgAN in the RCC setting [13,14]. Magyarlaki et al. reported IgAN in 11 out of 60 kidneys resected for RCC [15]. The pathophysiology has not been fully elucidated. However, Mimura et al. hypothesized that infiltrating lymphocytes and plasma cells around RCC produce IgA that likely contributes to mesangial deposition [16]. Interestingly, it has also been demonstrated that paraneoplastic IgAN can resolve following nephrectomy for RCC [17]. In this patient, her IgAN was found concomitantly with a large renal mass, thus raising the question of whether her glomerular disease was a paraneoplastic process. While a biopsy of the mass was inconclusive, there was a high index of suspicion based on MRI imaging that this was a cystic RCC. No consensus guideline exists recommending malignancy screening following all new cases of IgAN. The presence of malignancy should be strongly considered if the patient has risk factors, including advanced age, smoking status, and history of GI or respiratory mucosal cancers [18]. Clinical judgment and the overall clinical picture should ultimately guide focused screening efforts.

## Conclusions

This case highlights a rare presentation of IgAN in the context of a large renal mass, raising suspicion for a potential paraneoplastic phenomenon. It emphasizes the importance of considering malignancy in secondary IgAN, particularly in patients with risk factors such as advanced age and a suspicious renal mass. A multidisciplinary approach remains essential for accurate diagnosis and management, with further research needed to clarify the link between IgAN and malignancy.
